# Donor–recipient Hepatitis B serological patterns and post-transplant HBV events in pediatric living donor liver transplantation: a single-center retrospective cohort with a nested case series

**DOI:** 10.3389/fimmu.2026.1977829

**Published:** 2026-10-12

**Authors:** Ying Le, Haifeng Tang, Quanhai Zhang, Jiajun Weng, Lianbin Huang, Xiaoke Dai, Mingman Zhang

**Affiliations:** 1 Department of Hepatobiliary Surgery Children’s Hospital of Chongqing Medical University, National Clinical Research Center for Child Health and Disorders, Ministry of Education Key Laboratory of Child Development and Disorders, Chongqing Key Laboratory of Pediatric Metabolism and Inflammatory Diseases, Chongqing, China; 2 The Second Affiliated Hospital of Chongqing Medical University, Chongqing, China; 3 Baoshenghu Community Health Service Center, Liangjia, Chongqing, China

**Keywords:** anti-HBc, anti-HBs, competing risks, Fine–Gray regression, hepatitis B virus, living donor liver transplantation, pediatric liver transplantation

## Abstract

**Background:**

The combined effects of donor hepatitis B core antibody (anti-HBc) status and recipient pre-transplant hepatitis B surface antibody (anti-HBs) levels on post-transplant HBV events remain unclear in pediatric living donor liver transplantation (LDLT).

**Methods:**

We retrospectively analyzed pediatric LDLT recipients transplanted between May 2018 and June 2023. The primary outcome was the first post-transplant hepatitis B surface antigen (HBsAg)-positive result, with death treated as a competing event. Recipients were stratified by donor anti-HBc status and recipient anti-HBs level (≥100 vs <100 mIU/mL). Antiviral prophylaxis with entecavir (0.015 mg/kg once daily) was given only to recipients of anti-HBc-positive grafts, and hepatitis B immune globulin was not used. Cumulative incidence was compared using Gray’s test, and associated factors were evaluated using Fine–Gray regression.

**Results:**

Among 229 recipients, 14 (6.1%) developed HBV events. Events occurred in 0 of 97 recipients with an anti-HBc-negative donor and anti-HBs ≥100 mIU/mL (G1), 3 of 67 with an anti-HBc-negative donor and anti-HBs <100 mIU/mL (G2), 2 of 29 with an anti-HBc-positive donor and anti-HBs ≥100 mIU/mL (G3), and 9 of 36 with an anti-HBc-positive donor and anti-HBs <100 mIU/mL (G4); the corresponding 5-year cumulative incidences were 0%, 4.5%, 6.9% and 25.0% (global Gray’s test, P<0.001). Donor anti-HBc positivity (sHR, 8.28; 95% CI, 2.27–30.28; P = 0.001) and recipient anti-HBs <100 mIU/mL (sHR, 6.02; 95% CI, 1.32–27.55; P = 0.021) were independently associated with HBV events, although the wide confidence intervals reflect the small number of events (n = 14) and the magnitude of these estimates should be interpreted with caution.

**Conclusions:**

Donor anti-HBc positivity and low recipient pre-transplant anti-HBs were independently associated with post-transplant HBV events after pediatric LDLT. Combined donor–recipient serological stratification identified a clear risk gradient and may support risk-adapted HBV surveillance and prevention.

## Introduction

Living donor liver transplantation (LDLT) is an important treatment option for children with end-stage liver disease, particularly in regions where deceased-donor organs remain limited (1–3). In pediatric LDLT, parents constitute the predominant donor source, making the appropriate use of otherwise suitable donors with serological evidence of previous hepatitis B virus (HBV) exposure an important clinical consideration.

Liver grafts from hepatitis B core antibody (anti-HBc)-positive donors may harbor persistent intrahepatic HBV despite the absence of circulating hepatitis B surface antigen (HBsAg) (4, 5); in areas with a high prevalence of anti-HBc positivity, *de novo* hepatitis B after transplantation is correspondingly more common (6). Under post-transplant immunosuppression, such latent viral reservoirs may contribute to post-transplant HBV events. Recipient immunity may modify this risk, with hepatitis B surface antibody (anti-HBs) generally considered protective (7). However, the level of anti-HBs required to provide clinically meaningful protection in immunosuppressed pediatric recipients remains uncertain (8, 9), and donor and recipient serological factors are frequently considered independently rather than jointly (10).

Pediatric recipients also present specific challenges for interpretation of HBV serology. Most pediatric LDLT recipients are infants, in whom pre-transplant anti-HBc positivity may reflect passively transferred maternal immunoglobulin rather than previous HBV infection (11, 12). Furthermore, when the mother serves as the living donor, maternal and donor HBV serological profiles are inherently linked (11). These features complicate source attribution when HBsAg positivity subsequently develops after transplantation.

Competing events represent another methodological consideration. Death before development of an HBV event prevents subsequent observation of the primary outcome and should therefore be incorporated into time-to-event analyses rather than treated as ordinary censoring (13).

In this study, we evaluated the association of donor anti-HBc status and recipient pre-transplant anti-HBs levels with post-transplant HBV events in a single-center cohort of pediatric LDLT recipients using competing-risk methods. We further examined the joint donor–recipient serological pattern and performed a nested descriptive case series of recipients who developed HBV events to characterize their subsequent virological trajectories and exploratory serological attribution patterns.

## Methods

### Study design and participants

We conducted a single-center retrospective cohort study of pediatric recipients who underwent LDLT between May 2018 and June 2023 at the Children’s Hospital of Chongqing Medical University, Chongqing, China. All consecutive pediatric LDLT recipients during the study period were screened for eligibility.

All consecutive pediatric recipients (aged <18 years) who underwent living-donor liver transplantation at our center between May 2018 and June 2023 were screened for eligibility. Recipients who were lost to follow-up and for whom post-transplant HBV outcome status could not be adequately ascertained were excluded. All donors and all recipients were documented as HBsAg-negative before transplantation. Of 237 recipients initially identified, eight were excluded because of loss to follow-up, leaving 229 recipients in the final analytical cohort (**Figure 1**).

**Figure 1:**
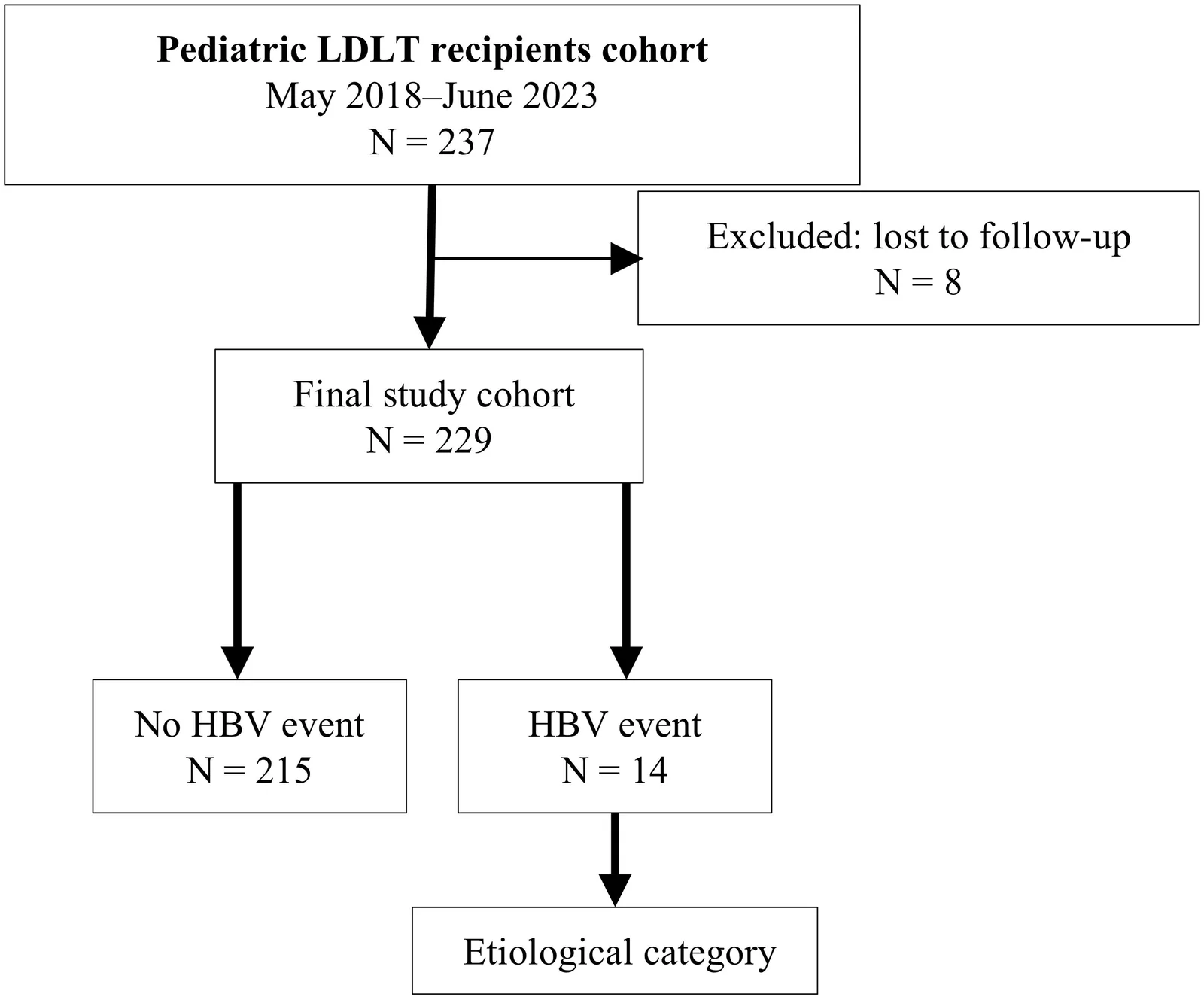
Study flow diagram. Among 237 pediatric recipients who underwent LDLT between May 2018 and June 2023, eight were excluded because of loss to follow-up. The final analytical cohort comprised 229 recipients, of whom 14 developed a post-transplant HBV event and 215 did not. LDLT, living donor liver transplantation; HBV, hepatitis B virus.

Clinical, demographic, transplantation, and laboratory data were retrospectively obtained from electronic medical records.

The study protocol was reviewed and approved by the Ethics Committee of the Children’s Hospital of Chongqing Medical University [Approval No. (2026) Lun Shen (Lin Yan) No. (268)]. The requirement for individual patient informed consent was waived because of the retrospective observational design and use of anonymized clinical data. All living-donor organs were obtained through voluntary donation with written informed consent in accordance with applicable national regulations and the principles of the Declaration of Helsinki.

### HBV prophylaxis and post-transplant surveillance

Perioperative and post-transplant HBV prevention followed the institutional protocol in use at our center during the study period. Consistent with Chinese national immunization requirements, all recipients were scheduled to receive the standard hepatitis B vaccination series (three doses at 0, 1 and 6 months, with the first dose administered within 24 h of birth) before transplantation (14). However, because the majority of recipients were young infants who underwent urgent living-donor liver transplantation, most had received only the first dose, and the extent to which the full series had been completed before transplantation could not be retrospectively verified (15, 16).

Hepatitis B immune globulin was not administered in this cohort (17). Post-transplant antiviral prophylaxis was restricted to recipients of anti-HBc-positive grafts (G3 and G4; n = 65), who received entecavir 0.015 mg/kg once daily throughout the perioperative period, on the basis that anti-HBc-positive grafts carry a recognized risk of *de novo* HBV infection (18). Recipients of anti-HBc-negative grafts (G1 and G2; n = 164) did not receive antiviral prophylaxis. Recipients who developed an HBV event began entecavir at 0.015 mg/kg once daily from the date of the first documented HBsAg-positive result. Antiviral prophylaxis was therefore determined by donor anti-HBc status, but was not modified according to recipient pre-transplant anti-HBs level.

### HBV serological variables and risk-group definitions

Serum HBsAg, anti-HBs and anti-HBc were measured on an Abbott ARCHITECT i2000SR automated immunoassay analyzer and on a Johnson & Johnson Vitros line using chemiluminescent immunoassay (CLIA). HBsAg is reported in IU/mL and anti-HBs in mIU/mL; anti-HBs values recorded in IU/L were converted according to the international standard. HBeAg, anti-HBe and anti-HBc were determined qualitatively and are expressed as signal-to-cutoff (s/co) ratios. HBV DNA was quantified by real-time fluorescence-based polymerase chain reaction (PCR) using a SLAN-96S automated medical PCR analysis system (Shanghai Hongshi Medical Technology Co., Ltd.) and is reported in IU/mL, with a lower limit of detection of 400 IU/mL.

Donor anti-HBc status was classified as positive or negative according to pre-transplant serological testing. Recipient pre-transplant anti-HBs concentration was analyzed as both a continuous variable and a dichotomous variable using a prespecified cutoff of 100 mIU/mL. Anti-HBs concentrations ≥100 mIU/mL were classified as high and concentrations <100 mIU/mL as low (8, 9).

Recipients were assigned to four donor–recipient serological groups:

G1 comprised recipients with an anti-HBc-negative donor and recipient anti-HBs ≥100 mIU/mL.
G2 comprised recipients with an anti-HBc-negative donor and recipient anti-HBs <100 mIU/mL.
G3 comprised recipients with an anti-HBc-positive donor and recipient anti-HBs ≥100 mIU/mL.
G4 comprised recipients with an anti-HBc-positive donor and recipient anti-HBs <100 mIU/mL.

Recipient pre-transplant anti-HBc status was additionally recorded as a baseline serological characteristic. Because maternal IgG crosses the placenta and may persist through infancy, both a positive anti-HBc result and, to a lesser degree, a detectable anti-HBs concentration in an infant recipient may reflect passively acquired maternal antibody rather than the child’s own infection or immune status. This is of particular relevance when the mother was also the living donor, in which case the maternal and donor serological profiles are inherently linked. Recipient anti-HBc status was therefore not included in the primary multivariable model, and the same consideration was applied when the joint donor–recipient serological patterns were interpreted.

### Outcome definition and follow-up

The primary outcome was a post-transplant HBV event, defined as the first documented HBsAg-positive result after LDLT. Because HBsAg positivity triggered confirmatory testing in this cohort, HBV DNA and liver biochemistry were measured at the time of the first HBsAg-positive result where available and were used to support, but not to define, the outcome. Events were further characterized according to the duration of HBsAg positivity, the HBV DNA result and the pre-transplant serological profile, so as to distinguish transient antigenemia from sustained infection.

Follow-up began on the date of transplantation and continued until the first HBV event, death, the last documented clinical assessment, or the administrative end of follow-up, as applicable. Death occurring before an HBV event was treated as a competing event. Recipients who remained alive without an HBV event were censored at their last observed follow-up.

When an HBV event and death occurred on the same recorded date, the recipient was classified as having experienced an HBV event if HBsAg positivity had been documented before or at the assessment preceding death.

Among recipients who developed an HBV event, subsequent HBsAg results were reviewed. Documented HBsAg clearance was defined as the first subsequent negative HBsAg result after the initial positive result. Recipients without a documented negative HBsAg result by their final available assessment were classified as having persistent HBsAg positivity.

### Nested case-series assessment

Recipients who developed a post-transplant HBV event constituted a nested descriptive case series. Individual trajectories from transplantation to the final assessment were reviewed together with recipient age, donor–recipient relationship, donor and recipient pre-transplant HBV serology, and available maternal HBV serology. On the basis of these profiles, cases were categorized into five prespecified exploratory attribution patterns: A, a graft-associated pattern (donor anti-HBc-positive with recipient pre-transplant anti-HBc-negative); B, a graft-associated pattern with possible passive maternal anti-HBc confounding (an anti-HBc-positive mother serving as the donor, with an anti-HBc-positive infant recipient); C, a recipient-side or maternal signal in the presence of an anti-HBc-negative donor; D, indeterminate dual-source attribution; and E, a pattern unexplained by donor or recipient serology (both anti-HBc-negative). Because donor–recipient viral sequence analysis was unavailable, these categories were descriptive and hypothesis-generating and were not used as predictors or outcomes in the primary statistical analyses.

### Statistical analysis

Continuous variables were summarized as medians and interquartile ranges (IQRs), and categorical variables as counts and percentages. Baseline characteristics were compared between recipients with and without post-transplant HBV events. Continuous variables were compared using the Mann–Whitney U test and categorical variables using Fisher’s exact test. Because only 14 HBV events occurred, baseline P values were considered descriptive.

The cumulative incidence function of post-transplant HBV events was estimated using the Aalen–Johansen estimator, with death before an HBV event treated as a competing event. Cumulative incidence functions were evaluated according to donor anti-HBc status, recipient anti-HBs status dichotomized at 100 mIU/mL, and the four joint donor–recipient serological groups.

Equality of cumulative incidence functions was assessed using Gray’s test. Cumulative incidence estimates with 95% confidence intervals (CIs) were reported at 1, 2, 3, and 5 years after transplantation.

Following a significant global Gray’s test across the four donor–recipient serological groups, all six pairwise Gray’s tests were performed. To account for multiple comparisons, P values were adjusted using the Holm procedure. Both unadjusted and Holm-adjusted P values are presented in **Supplementary Table S2**, with statistical inference based primarily on the adjusted P values.

Fine–Gray proportional subdistribution hazards regression was used to evaluate associations between baseline serological factors and the cumulative incidence of HBV events while accounting for death as a competing event (19). Separate univariable models were fitted for donor anti-HBc positivity and recipient anti-HBs <100 mIU/mL. A multivariable model subsequently included both variables simultaneously.

Associations were expressed as subdistribution hazard ratios (sHRs) with 95% CIs and two-sided P values. Given the limited number of HBV events, the multivariable model was intentionally restricted to these two prespecified serological predictors to limit model overfitting.

Overall survival was estimated by the Kaplan–Meier method, with recipients alive at the last documented assessment censored at that date. Because only 12 deaths occurred, the survival estimates are descriptive and no between-group test was performed. Given the small number of events (n = 14), the point estimates from the Fine–Gray models are imprecise; the results are therefore interpreted in terms of the direction and consistency of the associations rather than the magnitude of the subdistribution hazard ratios. Statistical analyses were performed using R version 4.5.1.

## Results

### Study population

A total of 237 pediatric LDLT recipients were screened. Eight were excluded because of loss to follow-up, resulting in a final cohort of 229 recipients (**Figure 1**). During follow-up, 14 recipients developed a post-transplant HBV event and 215 did not, corresponding to a crude event proportion of 6.1%.

The median duration of follow-up was 4.7 years (IQR, 3.5-6.0 years). A total of 11 recipients died before experiencing an HBV event and were therefore treated as competing events in the cumulative incidence analyses.

### Baseline characteristics

The median age at transplantation was 5.0 months (IQR, 4.0–6.0 months), and 203 recipients (88.7%) were aged ≤12 months. Overall, 118 recipients (51.5%) were male. Biliary atresia was the predominant indication for transplantation, accounting for 203 recipients (88.6%). The median Pediatric End-Stage Liver Disease score at transplantation was 19.0 (IQR, 14.0–24.0).

The median donor age was 29.0 years (IQR, 26.0–34.0 years). Mothers were the living donors for 138 recipients (60.3%), and fathers were the donors for 91 (39.7%).

Recipients who subsequently developed an HBV event did not differ significantly from those without an HBV event with respect to age at transplantation, sex, primary indication for transplantation, PELD score, donor age, or donor–recipient relationship (**Table 1**).

**Table 1:** Baseline characteristics of 229 pediatric LDLT recipients, stratified by post-transplant HBV event status.

Characteristic	Total (n = 229)	HBV event (n = 14)	No HBV event (n = 215)	P value
Recipient characteristics				
Age at transplant, months, median (IQR)	5.00 (4.00, 6.00)	5.00 (5.00, 6.00)	5.00 (4.00, 6.00)	0.661
Age ≤12 months, n (%)	203 (88.65)	11 (78.57)	192 (89.30)	
Male sex, n (%)	118 (51.53)	6 (42.86)	112 (52.09)	0.503
Primary indication for LT, n (%)				0.144
Biliary atresia	203 (88.6)	11 (78.6)	192 (89.3)	
Metabolic disease	12 (5.2)	2 (14.3)	10 (4.7)	
Vascular / portal cavernoma	7 (3.1)	0	7 (3.3)	
Acute liver failure	1 (0.4)	0	1 (0.5)	
Alagille syndrome	2 (0.9)	1 (7.1)	1 (0.5)	
Caroli disease	3 (1.3)	0	3 (1.4)	
Langerhans cell histiocytosis	1 (0.4)	0	1 (0.5)	
PELD score at transplant, median (IQR)	19.00 (14.00, 24.00)	19.00 (14.00, 23.75)	19.00 (14.00, 24.00)	0.729
Donor characteristics				
Donor age, years, median (IQR)	29.00 (26.00, 34.00)	28.00 (26.25, 35.00)	29.00 (26.00, 34.00)	0.935
Donor-recipient relationship, n (%)				0.378
Mother	138 (60.26)	10 (71.43)	128 (59.53)	
Father	91 (39.74)	4 (28.57)	87 (40.47)	
Pre-transplant HBV serology				
Donor HBcAb positive, n (%)	65 (28.4)	12 (85.7)	53 (24.7)	<0.001
Recipient HBsAb, IU/L, median (IQR)	79.79 (19.46, 238.82)	31.09 (1.38, 62.70)	103.13 (19.99, 245.15)	0.024
Recipient HBsAb <100 IU/mL, n (%)	103 (45.0)	12 (85.71)	91 (42.33)	0.002
Recipient HBcAb positive, n (%)	25 (10.96)	6 (42.86)	19 (8.88)	<0.001

Data are presented as median (IQR) or n (%). P values were calculated using Mann-Whitney U test for continuous variables and Fisher's exact test for categorical variables. Given the small number of events (n=14), P values are reported for descriptive purposes only. Abbreviations: PELD, Pediatric End-Stage Liver Disease; GRWR, graft-to-recipient weight ratio; HBsAb, hepatitis B surface antibody; HBcAb, hepatitis B core antibody; LT, liver transplantation.

In contrast, marked differences were observed in donor and recipient HBV serological characteristics. Donor anti-HBc positivity was present in 12 of 14 recipients with an HBV event (85.7%) compared with 53 of 215 recipients without an HBV event (24.7%; P<0.001).

Recipients who developed an HBV event had lower pre-transplant anti-HBs concentrations than those without an event, with median concentrations of 31.09 mIU/mL (IQR, 1.38–62.70) and 103.13 mIU/mL (IQR, 19.99–245.15), respectively (P = 0.024). Pre-transplant anti-HBs <100 mIU/mL was present in 12 of 14 recipients with an HBV event (85.7%) and 91 of 215 recipients without an event (42.3%; P = 0.002).

Recipient pre-transplant anti-HBc positivity was also more frequent among recipients who developed an HBV event than among those who did not (42.9% [6/14] vs 8.8% [19/215]; P<0.001). Given the young age of most recipients and the possibility of passive maternal antibody transfer, this finding was considered descriptive and recipient anti-HBc status was not included in the primary multivariable model.

All 14 events were asymptomatic: none of the recipients had abdominal pain, jaundice or other clinical manifestations of hepatitis at the time of the first HBsAg-positive result. Event-time ALT ranged from 15 to 123 U/L and AST from 27 to 133 U/L. HBV DNA was available for 11 of the 14 recipients and was detectable in all 11 (range, 1.93 × 10^3^ to 3.74 × 10^8^ IU/mL) (**Supplementary Table S1**). All 14 recipients were already HBsAg-negative and had undetectable HBV DNA before transplantation, indicating that the events arose after transplantation rather than reflecting pre-existing viremia. None of the 14 recipients who developed an HBV event had received treatment for acute rejection in the period preceding the event.

### HBV events according to joint donor–recipient serological pattern

The crude frequency of HBV events differed substantially across the four donor–recipient serological groups (**Table 2**).

**Table 2:** HBV event risk stratified by donor HBcAb and recipient pre-transplant HBsAb status.

Donor HBcAb	Recipient HBsAb ≥100 IU/mL	Recipient HBsAb <100 IU/mL
Negative	0 / 97 (0%)	3 / 67 (4.5%)
Positive	2 / 29 (6.9%)	9 / 36 (25.0%)

Data are presented as number of HBV events / total number of recipients in the subgroup (%). HBV event was defined as post-transplant HBsAg positivity. HBsAb, hepatitis B surface antibody; HBcAb, hepatitis B core antibody.

No HBV events occurred among the 97 recipients in G1. Among recipients with anti-HBc-negative donors, HBV events occurred in 3 of 67 recipients with anti-HBs <100 mIU/mL (G2; 4.5%). Among recipients with anti-HBc-positive donors, events occurred in 2 of 29 recipients with anti-HBs ≥100 mIU/mL (G3; 6.9%) and 9 of 36 recipients with anti-HBs <100 mIU/mL (G4; 25.0%).

Thus, the highest crude event proportion was observed among recipients with the combination of donor anti-HBc positivity and recipient pre-transplant anti-HBs <100 mIU/mL.

### Cumulative incidence of HBV events

The cumulative incidence of post-transplant HBV events was higher among recipients of grafts from anti-HBc-positive donors than among recipients of grafts from anti-HBc-negative donors (Gray’s test, P<0.001; **Figure 2A**).

**Figure 2:**
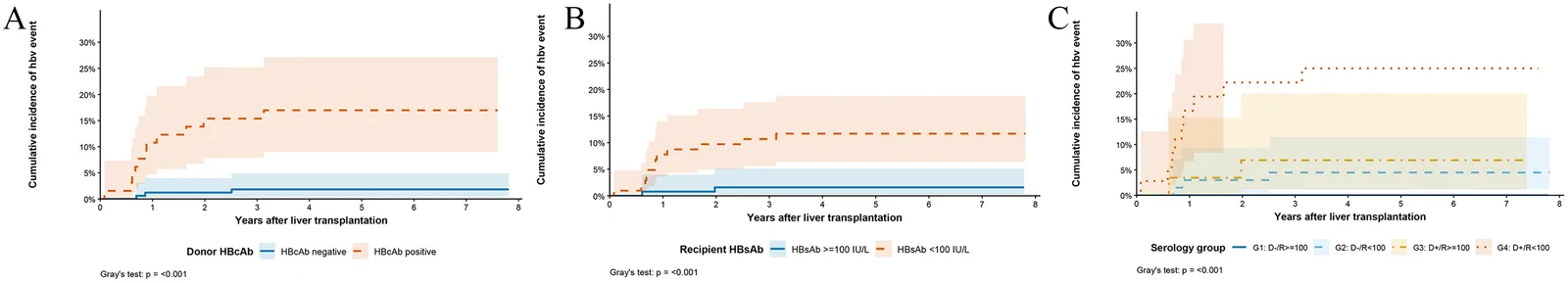
Cumulative incidence of post-transplant HBV events according to donor and recipient HBV serological status. Cumulative incidence functions were estimated using the Aalen–Johansen method with death before an HBV event treated as a competing event. **(A)** Donor anti-HBc-positive versus anti-HBc-negative status. **(B)** Recipient pre-transplant anti-HBs <100 versus ≥100 mIU/mL. **(C)** Joint donor–recipient serological groups: G1, donor anti-HBc negative/recipient anti-HBs ≥100 mIU/mL; G2, donor anti-HBc negative/recipient anti-HBs <100 mIU/mL; G3, donor anti-HBc positive/recipient anti-HBs ≥100 mIU/mL; and G4, donor anti-HBc positive/recipient anti-HBs <100 mIU/mL. Shaded areas represent 95% confidence intervals. Global comparisons were performed using Gray’s test.

Recipients with pre-transplant anti-HBs <100 mIU/mL similarly had a higher cumulative incidence of HBV events than those with anti-HBs ≥100 mIU/mL (Gray’s test, P<0.001; **Figure 2B**).

When donor anti-HBc and recipient anti-HBs status were considered jointly, cumulative incidence functions differed across the four serological groups (global Gray’s test, P<0.001; **Figure 2C** and **Table 3**). G4 consistently demonstrated the highest cumulative incidence, whereas no HBV events were observed in G1 during follow-up.

**Table 3:** Cumulative incidence of HBV events at fixed time points by donor-recipient serological strata.

Group	n	1-year % (95% CI)	2-year % (95% CI)	3-year % (95% CI)	5-year % (95% CI)
G1: D− / R≥100	97	0.0 (CI not estimable)	0.0 (CI not estimable)	0.0 (CI not estimable)	0.0 (CI not estimable)
G2: D− / R<100	67	3.0 (0.6–9.3)	3.0 (0.6–9.3)	4.5 (1.2–11.4)	4.5 (1.2–11.4)
G3: D+ / R≥100	29	3.4 (0.2–15.3)	6.9 (1.2–20.1)	6.9 (1.2–20.1)	6.9 (1.2–20.1)
G4: D+ / R<100	36	16.7 (6.6–30.6)	22.2 (10.3–37.0)	22.2 (10.3–37.0)	25.0 (12.3–40.0)

Estimates were derived from the Aalen-Johansen estimator. The overall Gray’s test for equality of cumulative incidence functions across the four groups yielded P < 0.001. .

At 1 year after transplantation, the cumulative incidence was 0% in G1, 3.0% (95% CI, 0.6%–9.3%) in G2, 3.4% (95% CI, 0.2%–15.3%) in G3, and 16.7% (95% CI, 6.6%–30.6%) in G4.

At 2 years, the respective cumulative incidences were 0%, 3.0% (95% CI, 0.6%–9.3%), 6.9% (95% CI, 1.2%–20.1%), and 22.2% (95% CI, 10.3%–37.0%).

At 3 years, cumulative incidence was 0% in G1, 4.5% (95% CI, 1.2%–11.4%) in G2, 6.9% (95% CI, 1.2%–20.1%) in G3, and 22.2% (95% CI, 10.3%–37.0%) in G4.

By 5 years, cumulative incidence was 0% in G1, 4.5% (95% CI, 1.2%–11.4%) in G2, 6.9% (95% CI, 1.2%–20.1%) in G3, and 25.0% (95% CI, 12.3%–40.0%) in G4.

These results demonstrated a graded pattern across the joint donor–recipient serological strata. Importantly, this pattern should not be interpreted as evidence of a statistical interaction between donor anti-HBc and recipient anti-HBs because an interaction term was not formally tested.

### Causes of death among the competing events

Among the 229 recipients, 12 died during follow-up; 11 died before a post-transplant HBV event and were therefore treated as competing events. Causes of death were infection, sepsis or respiratory failure in 5 of 11 (45.5%), post-transplant lymphoproliferative disorder (PTLD) in 3 of 11 (27.3%), and graft failure or surgical/vascular complications in 3 of 11 (27.3%); in one recipient death occurred at another hospital and the cause could not be established (**Supplementary Table S3**).

### Survival by serological group

Overall survival did not differ substantially across the four donor–recipient serological groups, with 5-year Kaplan–Meier estimates of 96.9% in G1, 93.2% in G2, 89.7% in G3 and 94.2% in G4 (**Supplementary Table S4**).

### Pairwise comparisons of the four serological groups

Following Holm adjustment for multiple comparisons, significant differences in cumulative incidence remained between G1 and G3 (adjusted P = 0.036), G1 and G4 (adjusted P<0.006), and G2 and G4 (adjusted P = 0.010). The differences between G1 and G2, G2 and G3, and G3 and G4 were not statistically significant after adjustment (**Supplementary Table S2**). Notably, the nominal difference between G1 and G2 did not remain significant after correction for multiple comparisons.

### Fine–Gray regression analysis

In univariable Fine–Gray regression, donor anti-HBc positivity was associated with a higher subdistribution hazard of an HBV event than donor anti-HBc negativity (sHR, 9.98; 95% CI, 2.81–35.42; P<0.001). Recipient pre-transplant anti-HBs <100 mIU/mL was similarly associated with a higher subdistribution hazard than anti-HBs ≥100 mIU/mL (sHR, 7.66; 95% CI, 1.72–34.09; P = 0.007).

Both factors remained associated with HBV events when included simultaneously in the multivariable model. Donor anti-HBc positivity was associated with an adjusted sHR of 8.28 (95% CI, 2.27–30.28; P = 0.001), whereas recipient anti-HBs <100 mIU/mL was associated with an adjusted sHR of 6.02 (95% CI, 1.32–27.55; P = 0.021) (**Table 4**).

**Table 4:** Fine-gray regression analysis for post-transplant HBV events.

Variable	sHR (95% CI)	P value
Univariable models		
Donor HBcAb positive	9.98 (2.81–35.42)	<0.001
Recipient HBsAb <100 IU/mL	7.66 (1.72–34.09)	0.007
Multivariable model		
Donor HBcAb positive	8.28 (2.27–30.28)	0.001
Recipient HBsAb <100 IU/mL	6.02 (1.32–27.55)	0.021

The multivariable model included both donor HBcAb and recipient HBsAb status. sHR, subdistribution hazard ratio; CI, confidence interval.

The confidence intervals were wide, reflecting the limited number of HBV events, but the direction of association was consistent between the univariable and multivariable models, and the magnitude of the subdistribution hazard ratios should be interpreted with caution.

### Virological trajectories of recipients with HBV events

The individual virological trajectories of the 14 recipients who developed post-transplant HBsAg positivity are shown in **Figure 3**.

**Figure 3:**
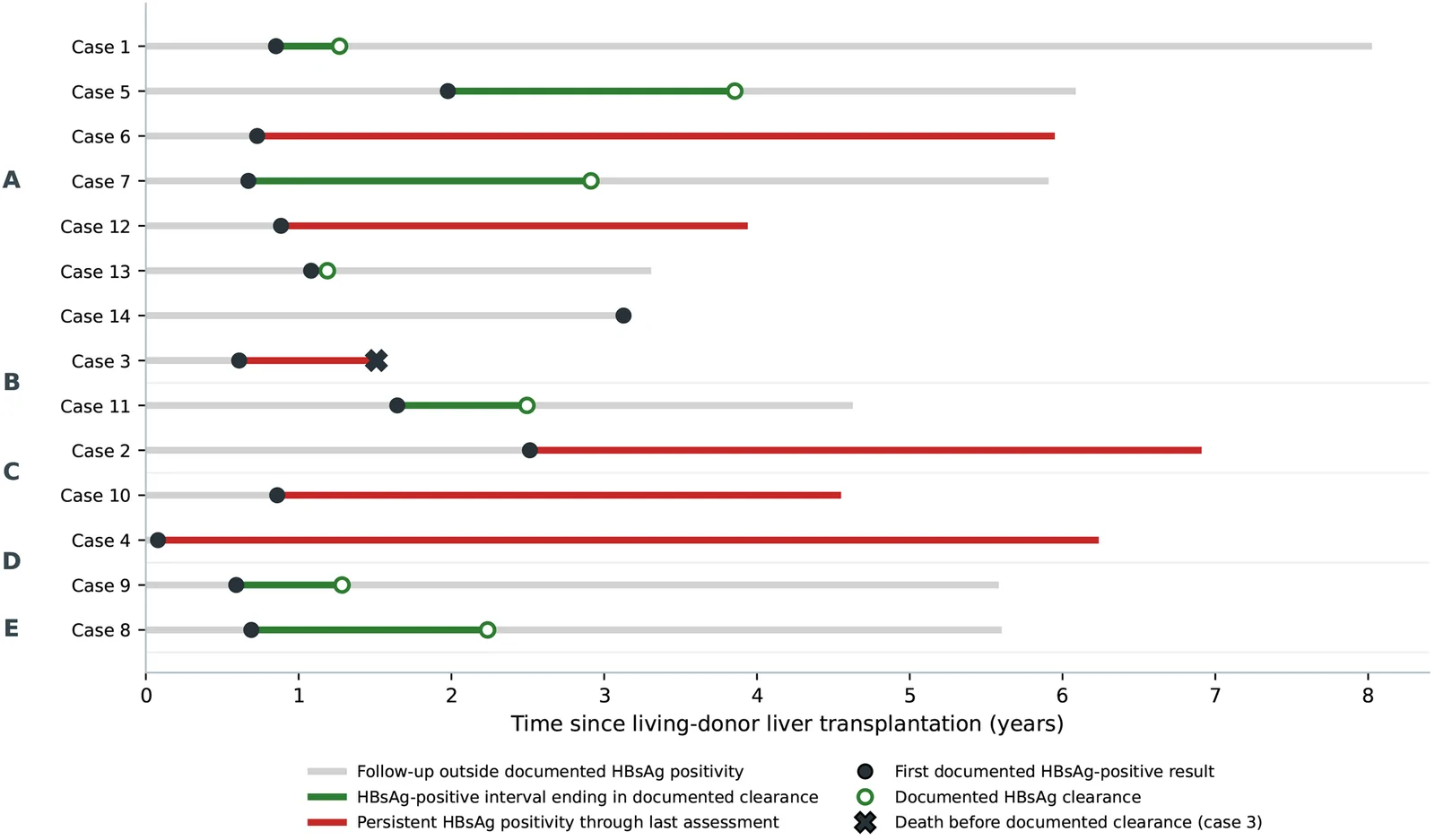
Virological trajectories and exploratory serological attribution patterns among the 14 recipients with post-transplant HBV events. Each horizontal line represents the follow-up trajectory of one recipient from transplantation to the final available assessment or death. Filled circles indicate the first documented post-transplant HBsAg-positive result. Open circles indicate documented HBsAg clearance. Green segments represent the interval from the initial HBsAg-positive result to documented clearance, red segments indicate persistent HBsAg positivity through the final assessment, and the dark segment terminating in an X indicates death before documented clearance. Gray segments represent follow-up outside the period of documented HBsAg positivity. Cases are grouped according to exploratory serological attribution patterns: **(A)** graft-associated serological pattern; **(B)** graft-associated pattern with potential passive maternal anti-HBc confounding; **(C)** recipient-side or maternal serological signal with an anti-HBc-negative donor; **(D)** indeterminate dual-source pattern; and **(E)** unexplained serological pattern. These patterns are descriptive and do not represent virologically confirmed transmission routes. Detailed case characteristics are provided in **Supplementary Table S1**.

The median time from transplantation to the first documented HBsAg-positive result was 312 days (IQR 245–601 days; range 29–1142 days); 9 of the 14 events occurred within the first year after transplantation and 2 occurred after more than 2 years.

Seven of the 14 recipients (50.0%) subsequently achieved documented HBsAg clearance. Six recipients (42.9%) remained HBsAg positive through their last available follow-up, and one recipient (7.1%) died without documented HBsAg clearance. Persistent HBsAg positivity also occurred over variable durations of follow-up, demonstrating substantial heterogeneity in the clinical course following an HBV event.

### Exploratory serological attribution patterns

Of the 14 recipients with HBV events, 7 were classified as pattern A, 2 as pattern B, 2 as pattern C, 2 as pattern D and 1 as pattern E (**Supplementary Table S1**). Maternal HBsAg positivity provided additional evidence of possible maternal or pre-transplant exposure in one of the two pattern C cases, and no plausible source could be inferred from serology in the single pattern E case. These categories describe serological patterns and do not represent virologically confirmed routes of transmission.

## Discussion

In this single-center cohort of 229 pediatric LDLT recipients, post-transplant HBV events occurred in 14 recipients. Three principal findings emerged. First, donor anti-HBc positivity was strongly associated with subsequent HBV events. Second, recipient pre-transplant anti-HBs <100 mIU/mL was independently associated with a higher cumulative incidence of HBV events. Third, joint consideration of these two serological characteristics identified a marked risk gradient: no events occurred among recipients with an anti-HBc-negative donor and anti-HBs ≥100 mIU/mL, whereas one-quarter of recipients with both donor anti-HBc positivity and anti-HBs <100 mIU/mL developed an HBV event.

The association between donor anti-HBc positivity and post-transplant HBsAg positivity is biologically plausible. Anti-HBc positivity reflects previous HBV exposure, and HBV covalently closed circular DNA may persist within hepatocytes despite the absence of detectable circulating HBsAg (4, 20, 21). The immunosuppressive environment after liver transplantation may permit renewed viral replication from such intrahepatic reservoirs (22). Our findings extend this concern to pediatric LDLT, in which parental living donors constitute the majority of available donors and exclusion of otherwise suitable anti-HBc-positive donors may substantially restrict donor availability.

Recipient humoral immunity appeared to modify the observed risk profile. Recipients with pre-transplant anti-HBs <100 mIU/mL had a substantially higher subdistribution hazard of HBV events than those with higher antibody concentrations (23). Nevertheless, anti-HBs ≥100 mIU/mL did not provide absolute protection: two events occurred among recipients of anti-HBc-positive grafts despite anti-HBs concentrations above this threshold (8, 9). Thus, anti-HBs should probably be viewed as one component of risk stratification rather than as a binary marker of complete protection.

Joint stratification provided additional clinically interpretable information. The lowest-risk pattern was observed in G1, in which no HBV events occurred, whereas G4 had a 5-year cumulative incidence of 25.0%. G2 and G3 demonstrated intermediate cumulative incidences, consistent with prior reports in pediatric and adult LDLT cohorts (24–26). This graded distribution suggests that both graft-related HBV exposure and recipient antibody status contribute to the observed post-transplant risk. However, because a formal statistical interaction between donor anti-HBc and recipient anti-HBs was not tested, these findings should not be interpreted as demonstrating biological or statistical interaction.

Our findings are consistent with previous pediatric and adult LDLT series of anti-HBc-positive grafts, which have reported that donor anti-HBc positivity confers a substantial risk of *de novo* HBV infection while recipient anti-HBs appears partially protective (7, 23–26). Unlike most earlier reports, in which recipients were predominantly adults receiving deceased-donor grafts, the present cohort consisted almost entirely of infants receiving parental living-donor grafts, a setting in which the exclusion of anti-HBc-positive donors would substantially reduce donor availability. The absence of any event among recipients with an anti-HBc-negative donor and anti-HBs ≥100 mIU/mL is likewise in keeping with the low risk reported for this combination.

Recipient pre-transplant anti-HBc positivity was substantially more common among recipients who subsequently developed HBV events. Interpretation of this association is particularly challenging in the present pediatric cohort because almost 90% of recipients were ≤12 months old. Maternal IgG anti-HBc can cross the placenta and persist during infancy; consequently, a positive pre-transplant anti-HBc result in a young child does not necessarily demonstrate previous HBV infection. This was the principal reason that recipient anti-HBc status was not incorporated into the primary multivariable model.

The nested case series further illustrates this interpretive complexity. Seven of 14 event cases displayed a relatively straightforward donor-positive/recipient-negative anti-HBc pattern compatible with a graft-associated source. Other cases, however, demonstrated overlapping donor, recipient, and maternal serological findings. In infants receiving maternal grafts, recipient anti-HBc positivity may reflect passive maternal antibody, whereas in recipients of paternal grafts, independent maternal HBV exposure may provide an alternative explanation. One case occurred despite both donor and recipient being anti-HBc negative. Because donor–recipient viral sequence analysis was unavailable, these classifications were descriptive and hypothesis-generating and were not used as predictors or outcomes in the primary statistical analyses. Serology alone cannot establish the source of individual post-transplant HBV events (27). Viral sequencing or other molecular linkage approaches would be required for definitive source attribution (4, 20).

HBsAg clearance was documented in 7 of the 14 recipients (50.0%), with a median time to clearance of 310 days (range 39–819 days). Several factors may account for this heterogeneity. All recipients who developed an event began entecavir at the time of detection, and in at least one instance antigen clearance was accompanied by HBeAg loss and a rise in anti-HBc, consistent with antiviral suppression of a newly established infection. Younger age and a correspondingly greater degree of immunological naivety may favor a tolerant, high-replication state with persistent antigenemia, whereas older children — as illustrated by the three recipients aged 82–132 months in this series — appeared more likely to clear antigen. Age at transplantation did not differ between recipients who did and did not clear HBsAg, and larger cohorts will be required to identify the determinants of outcome.

The clinical implications of these findings are primarily risk-stratification oriented. Recipients with both an anti-HBc-positive donor and low pre-transplant anti-HBs constituted the highest-risk subgroup in this cohort and may warrant particularly close HBV surveillance and optimization of preventive strategies (28, 29). The present observational data, however, should not by themselves be used to define a specific antiviral prophylaxis regimen or to exclude anti-HBc-positive living donors. Notably, antiviral monotherapy without hepatitis B immune globulin is consistent with recent reports of a low frequency of HBsAg reemergence after withdrawal of hepatitis B immune globulin (30). Evaluation of prophylactic treatment must take into account the institutional vaccination, hepatitis B immune globulin, antiviral, and monitoring protocols applied during the study period (31–35).

With an overall event proportion of 6.1%, the present data do not support universal antiviral prophylaxis in pediatric LDLT. They instead support risk-adapted management. Recipients of anti-HBc-positive grafts with low pre-transplant anti-HBs constituted the highest-risk subgroup and may warrant the most intensive surveillance, whereas recipients with an anti-HBc-negative donor and anti-HBs ≥100 mIU/mL had no events during follow-up and may require less frequent monitoring. Because prophylaxis in this cohort was confined to recipients of anti-HBc-positive grafts, and because events still occurred in that group, whether prophylaxis should be intensified — by extending antiviral therapy, by adding hepatitis B immune globulin, or by active immunization — remains an open question that can only be answered prospectively.

Several limitations should be considered. First, this was a retrospective single-center study, which limits generalizability. Second, only 14 HBV events occurred, resulting in wide confidence intervals and limiting the number of variables that could be incorporated into multivariable analysis. Third, residual confounding by factors such as immunosuppressive exposure, vaccination status, anti-HBs dynamics after transplantation, antiviral prophylaxis, and intensity of HBV surveillance is possible. Fourth, the primary outcome was defined by documented HBsAg positivity; without systematic HBV DNA confirmation for every event, the study describes HBV serological events rather than molecularly confirmed transmission in all cases. Fifth, the timing of event detection depended on the clinical testing schedule and may therefore be subject to interval-censoring or surveillance-frequency effects. Sixth, maternal serological information was incomplete or nonspecific in some cases, and donor–recipient viral sequencing was unavailable, precluding definitive source attribution. Seventh, the 100-mIU/mL anti-HBs threshold should be externally validated rather than interpreted as a universally established protective cutoff. Eighth, immunosuppressive regimens and drug exposure were not recorded in a form suitable for analysis, so we could not determine whether the intensity of immunosuppression differed between serological groups, and this remains a potential source of residual confounding. Ninth, HBV DNA was not measured at the time of every event, and anti-HBs concentrations were recorded in both mIU/mL and IU/L in the source records and were harmonized for analysis; standardized prospective measurement is desirable.

Despite these limitations, the study has several strengths. It evaluates a well-defined pediatric LDLT cohort in which parental living donation predominated, incorporates death appropriately as a competing event, evaluates donor and recipient serology jointly rather than in isolation, and complements cohort-level risk estimates with detailed case-level virological trajectories. Because antiviral prophylaxis was given only to recipients of anti-HBc-positive grafts, the higher cumulative incidence observed in this group occurred despite prophylaxis; had prophylaxis been effective, this would have biased the association toward the null. In addition, within each prophylaxis stratum, the comparison between recipients with high and low pre-transplant anti-HBs was not confounded by antiviral exposure. Because hepatitis B immune globulin was not used and vaccination was frequently incomplete before transplantation, the generalizability of our prophylaxis-related conclusions is limited and should be considered when interpreting the risk gradient.

In conclusion, donor anti-HBc positivity and recipient pre-transplant anti-HBs <100 mIU/mL were independently associated with post-transplant HBV events after pediatric LDLT. Joint donor–recipient serological classification identified a clinically meaningful gradient of cumulative incidence, with the highest risk observed when an anti-HBc-positive graft was transplanted into a recipient with low anti-HBs. These findings support individualized HBV risk assessment and surveillance in pediatric LDLT and warrant confirmation in larger multicenter cohorts.

## Data Availability

The original contributions presented in the study are included in the article/**Supplementary Material**. Further inquiries can be directed to the corresponding author.
