# Supplementary material for: Donor–recipient Hepatitis B serological patterns and post-transplant HBV events in pediatric living donor liver transplantation: a single-center retrospective cohort with a nested case series
Source: Front Immunol. 2026 Oct 12;17:1977829. doi: 10.3389/fimmu.2026.1977829 (PMC13696473; doi:10.3389/fimmu.2026.1977829)
Supplement: Supplementary file 1 [file SupplementaryFile1.docx]

**Supplementary Table S1. Clinical, serological, biochemical and virological characteristics of the 14 recipients with post-transplant HBV events, and exploratory serological attribution patterns**

| **Case** | **Age (mo) / sex** | **Primary disease** | **Donor (relation)** | **Donor anti-HBc** | **Donor anti-HBs (mIU/mL)** | **Recipient pre-LT anti-HBc** | **Recipient pre-LT anti-HBs (mIU/mL)** | **Maternal HBV serology** | **Time to HBsAg+ (days)** | **HBV DNA (IU/mL)** | **ALT (U/L)** | **AST (U/L)** | **TBil (µmol/L)** | **HBsAg outcome** | **Attribution pattern** | **Rationale for classification** |
| --- | --- | --- | --- | --- | --- | --- | --- | --- | --- | --- | --- | --- | --- | --- | --- | --- |
| 1 | 6 / F | Biliary atresia | Mother | + | 46.35 | − | 31.34 | Same as donor | 311 | 1.93 × 10³ | 66.8 | 50.2 | 11.4 | Cleared (day 152) | A | Donor anti-HBc+, recipient anti-HBc− |
| 2 | 5 / F | Biliary atresia | Mother | − | 93.63 | + | 63.54 | Same as donor | 918 | >1 × 10⁸ | 37 | 50 | 6.7 | Persistent | C | Donor anti-HBc−, recipient anti-HBc+ |
| 3 | 5 / M | Biliary atresia | Mother | + | 18.26 | + | 702.19 | Same as donor | 223 | >1 × 10⁸ | 45.9 | 51 | 18 | Persistent; died before clearance | B | Mother was the donor and was anti-HBc+; infant anti-HBc+ |
| 4 | 5 / M | Biliary atresia | Father | + | 15.56 | + | 75.27 | History of HBV infection | 29 | 1.95 × 10⁷ | 34 | 40 | 2.5 | Persistent | D | Paternal donor anti-HBc+, infant anti-HBc+, maternal HBV history |
| 5 | 5 / F | Biliary atresia | Mother | + | 249.75 | − | 177.15 | Same as donor | 722 | 6.74 × 10⁵ | 54 | 63 | 10.3 | Cleared (day 686) | A | Donor anti-HBc+, recipient anti-HBc− |
| 6 | 82 / F | Urea cycle disorder | Mother | + | 11.12 | − | 30.84 | Same as donor | 266 | 3.8 × 10⁴ | 15 | 32 | 7.9 | Persistent | A | Older child; no maternal antibody interference |
| 7 | 5 / M | Biliary atresia | Mother | + | 282.38 | − | 2.06 | Same as donor | 245 | 2.36 × 10³ | 53.7 | 93.8 | 5.9 | Cleared (day 819) | A | Donor anti-HBc+, recipient anti-HBc− |
| 8 | 132 / F | Wilson’s disease | Mother | − | 5.03 | − | 1.15 | Same as donor | 252 | 2.81 × 10⁴ | 39 | 27 | 8.3 | Cleared (day 565) | E | Both donor and recipient anti-HBc−; older child |
| 9 | 4 / M | Biliary atresia | Father | + | 692.94 | + | 22.45 | HBsAg−, anti-HBs 310, anti-HBc+ | 216 | 1.77 × 10⁸ | 27 | 33 | 11.7 | Cleared (day 253) | D | Paternal donor anti-HBc+, infant anti-HBc+, maternal anti-HBc+ |
| 10 | 4 / F | Biliary atresia | Father | − | 0.22 | + | 60.16 | HBsAg+, anti-HBs 4.3, anti-HBc+ | 314 | 1.38 × 10⁵ | 18 | 48 | 4.9 | Persistent | C | Donor anti-HBc−, recipient anti-HBc+, maternal HBsAg+ |
| 11 | 4 / F | Biliary atresia | Mother | + | 2.22 | + | 52.16 | Same as donor | 601 | Not available | 18 | 32 | 6 | Cleared (day 310) | B | Mother was the donor and was anti-HBc+; infant anti-HBc+ |
| 12 | 6 / F | Biliary atresia | Mother | + | 595.75 | − | 0.88 | Same as donor | 323 | Not available | 19 | 44 | 4.3 | Persistent | A | Donor anti-HBc+, recipient anti-HBc− |
| 13 | 86 / F | Alagille syndrome | Mother | + | 572.84 | − | 0 | Same as donor | 395 | Not available | 35 | 42 | 2.1 | Cleared (day 39) | A | Older child; no maternal antibody interference |
| 14 | 6 / M | Biliary atresia | Father | + | 212.91 | − | 0.2 | Reported HBV-negative | 1142 | 3.74 × 10⁸ | 123 | 133 | 8.9 | Persistent | A | Donor anti-HBc+, recipient anti-HBc− |

**Notes:** Time to HBsAg+ is the interval from transplantation to the first documented HBsAg-positive result. ALT, AST, TBil and HBV DNA are event-time values. All 14 recipients were asymptomatic at the time of the first HBsAg-positive result. Recipients were treated with entecavir 0.015 mg/kg once daily from the date of the first HBsAg-positive result. Attribution patterns: A, graft-associated serological pattern (donor anti-HBc+ with recipient pre-transplant anti-HBc−); B, graft-associated pattern with possible passive maternal anti-HBc confounding; C, recipient-side or maternal serological signal with an anti-HBc-negative donor; D, indeterminate dual-source pattern; E, unexplained pattern (both donor and recipient anti-HBc−). For recipients whose mother was also the living donor, “same as donor” indicates that the maternal serological profile was identical to the donor profile. Maternal HBV serology was obtained from pre-transplant records or late-pregnancy screening; for father donors it was obtained separately from the available maternal records. LT, liver transplantation; TBil, total bilirubin; anti-HBc, hepatitis B core antibody; anti-HBs, hepatitis B surface antibody.

**Supplementary Table S2. Pairwise Gray’s Test Comparisons Between Four Serological Groups**

| **Comparison** | **Gray’s Test P Value** | **Holm-adjusted P Value** |
| --- | --- | --- |
| G1 vs. G2 | 0.036 | 0.108 |
| G1 vs. G3 | 0.009 | 0.036 |
| G1 vs. G4 | <0.001 | <0.006 |
| G2 vs. G3 | 0.619 | 0.619 |
| G2 vs. G4 | 0.002 | 0.01 |
| G3 vs. G4 | 0.056 | 0.112 |

**Notes:** Pairwise comparisons of cumulative incidence functions were performed using Gray’s test following a significant global Gray’s test. P values were adjusted for six pairwise comparisons using the Holm procedure. Adjusted P values <0.05 were considered statistically significant. G1, donor anti-HBc negative/recipient anti-HBs ≥100 mIU/mL; G2, donor anti-HBc negative/recipient anti-HBs <100 mIU/mL; G3, donor anti-HBc positive/recipient anti-HBs ≥100 mIU/mL; G4, donor anti-HBc positive/recipient anti-HBs <100 mIU/mL.

**Supplementary Table S3. Causes of death among the 11 recipients who died before a post-transplant HBV event (competing events)**

| **Cause of death category** | **n (%)** |
| --- | --- |
| Infection, sepsis or respiratory failure | 5 (45.5) |
| Post-transplant lymphoproliferative disorder (PTLD) | 3 (27.3) |
| Graft failure or surgical / vascular complications | 3 (27.3) |
| Total | 11 (100) |

**Notes:** Deaths occurring before a post-transplant HBV event were treated as competing events. Causes were classified from death records into the three categories shown; individual-level mapping of causes to participants is not presented. One death occurred at another hospital after oral medication and the cause could not be established; this death is included in the infection group. Among the 14 recipients with post-transplant HBV events, one died without documented HBsAg clearance; that death is not counted among the 11 competing events.

**Supplementary Table S4. Overall survival by donor–recipient serological group (Kaplan–Meier estimates)**

| **Group** | **n** | **Deaths, n** | **1-year OS, %** | **3-year OS, %** | **5-year OS, %** |
| --- | --- | --- | --- | --- | --- |
| G1: donor anti-HBc− / recipient anti-HBs ≥100 mIU/mL | 97 | 3 | 97.9 | 96.9 | 96.9 |
| G2: donor anti-HBc− / recipient anti-HBs <100 mIU/mL | 67 | 4 | 100.0 | 95.5 | 93.2 |
| G3: donor anti-HBc+ / recipient anti-HBs ≥100 mIU/mL | 29 | 3 | 93.1 | 89.7 | 89.7 |
| G4: donor anti-HBc+ / recipient anti-HBs <100 mIU/mL | 36 | 2 | 97.2 | 97.2 | 94.2 |
| All recipients | 229 | 12 | 97.8 | 95.6 | 94.4 |

**Notes:** OS, overall survival; anti-HBc, hepatitis B core antibody; anti-HBs, hepatitis B surface antibody. Estimates were obtained by the Kaplan–Meier method with recipients alive at the last documented assessment censored at that date. Estimates are descriptive; no between-group test is reported because of the small number of deaths (n = 12). Deaths include one recipient who died after a post-transplant HBV event; the remaining 11 deaths were competing events. Median follow-up was 4.7 years (IQR 3.5–6.0).
